# Critical Linkages Between Livestock Production, Livestock Trade and Potential Spread of Human African Trypanosomiasis in Uganda: Bioeconomic Herd Modeling and Livestock Trade Analysis

**DOI:** 10.3389/fvets.2021.611141

**Published:** 2021-07-26

**Authors:** Walter O. Okello, Ewan T. MacLeod, Dennis Muhanguzi, Charles Waiswa, Alexandra P. Shaw, Susan C. Welburn

**Affiliations:** ^1^Infection Medicine, Biomedical Sciences, Edinburgh Medical School, College of Medicine and Veterinary Medicine, University of Edinburgh, Edinburgh, United Kingdom; ^2^Land & Water Business Unit, Commonwealth Scientific and Industrial Research Organisation (CSIRO), Canberra, ACT, Australia; ^3^Department of Biomolecular and Biolaboratory Sciences, School of Biosecurity, Biotechnical and Laboratory Sciences, College of Veterinary Medicine Animal Resources and Biosecurity, Makerere University, Kampala, Uganda; ^4^The Coordinating Office for Control of Trypanosomiasis in Uganda (COCTU), Kampala, Uganda; ^5^Avia-GIS, Zoersel, Belgium; ^6^Zhejiang University-University of Edinburgh Institute, Zhejiang University School of Medicine, Zhejiang University, Haining, China

**Keywords:** HAT, economic drivers, bio-economic, herd modelling, value chain, Uganda

## Abstract

**Background:** Tsetse-transmitted human African trypanosomiasis (HAT) remains endemic in Uganda. The chronic form caused by *Trypanosoma brucei gambiense* (gHAT) is found in north-western Uganda, whereas the acute zoonotic form of the disease, caused by *T. b. brucei rhodesiense* (rHAT), occurs in the eastern region. Cattle is the major reservoir of rHAT in Uganda. These two forms of HAT are likely to converge resulting in a public health disaster. This study examines the intricate and intrinsic links between cattle herd dynamics, livestock trade and potential risk of spread of rHAT northwards.

**Methods:** A bio-economic cattle herd model was developed to simulate herd dynamics at the farm level. Semi-structured interviews (*n* = 310), focus group discussions (*n* = 9) and key informant interviews (*n* = 9) were used to evaluate livestock markets (*n* = 9) as part of the cattle supply chain analysis. The cattle market data was used for stochastic risk analysis.

**Results:** Cattle trade in eastern and northern Uganda is dominated by sale of draft and adult male cattle as well as exportation of young male cattle. The study found that the need to import draft cattle at the farm level was to cover deficits because of the herd structure, which is mostly geared towards animal traction. The importation and exportation of draft cattle and disposal of old adult male cattle formed the major basis of livestock movement and could result in the spread of rHAT northwards. The risk of rHAT infected cattle being introduced to northern Uganda from the eastern region via cattle trade was found to be high (i.e. probability of 1).

**Conclusion:** Through deterministic and stochastic modelling of cattle herd and cattle trade dynamics, this study identifies critical links between livestock production and trade as well as potential risk of rHAT spread in eastern and northern Uganda. The findings highlight the need for targeted and routine surveillance and control of zoonotic diseases such as rHAT.

## Introduction

Human African trypanosomiasis (HAT), which is also known as sleeping sickness, is vector borne endemic disease in Africa (1). There are two forms of the disease namely *rhodesiense* HAT (rHAT) and *gambiense* HAT (gHAT). The rHAT, which is also known as acute Rhodesian form of HAT, is caused by *Trypanosoma brucei rhodesiense* and is mostly found in East Africa (2). Wildlife and domestic animals, especially cattle, are the main reservoir of rHAT (3, 4). Humans get rHAT after being bitten by *Glossina*, the tsetse fly (5, 6). Cattle are not affected by rHAT and show no apparent clinical signs but are known to drive outbreaks (7, 8). The gHAT, which is also known as chronic Gambian form of HAT, is caused by *T. b. gambiense* and it is mostly found in western Africa (9). Humans get gHAT through human-tsetse contact. Unlike rHAT, gHAT is not known to have animal reservoirs (10).

Diagnosis of HAT is difficult especially in low resource setting and it involves clinical examination, mass screening, detection using whole blood card agglutination trypanosomiasis test (CATT), and identifying the stage of the disease via examination of cerebrospinal fluid after a lumbar puncture (11). After staging, each form of HAT requires different treatment at different stages (11). Therefore, diagnosis and treatment of HAT can prove to be challenging in scenarios where the two forms of the disease merge (12). Furthermore, the two forms of HAT cannot be distinguished morphologically; microscopic examination is still a major method of diagnosis in low resource setting (13).

Uganda is the only country in Africa with the two forms of HAT, with rHAT being endemic in south-east while gHAT is restricted to north-west part of the country (14). However, it has been reported that rHAT is moving northwards making the merger of the two forms of HAT eminent (15). The old rHAT foci have been in south-east Uganda particularly in Busia, Namutumba, Iganga, and Tororo districts. However, rHAT has moved to central parts of eastern Uganda particularly Soroti and Kaberamaido (9). It has been reported that between 39.5% (16) and 54% (17) of cattle involved in inter-district trade in Uganda had moved from rHAT endemic districts in the south east region into northwest and central regions that had not experienced the disease. Furthermore, cattle trade has been implicated to be responsible for the last rHAT epidemic in south-east Uganda (17). Consequently, it is important to understand the unintended consequences of economic activity on disease spread (18).

Farming and livestock trade, as economic activities, may generate disease risk due to the trade-off between effective disease control and return on investment on livestock production (19). The economic link between livestock production and market is demand and supply of agricultural and food commodities and services; understanding the economic drivers of the two systems is integral to understanding intra and inter-district spread of diseases including zoonoses (20). In livestock markets, there are three tiers of sale of cattle and related agricultural inputs in developing countries (21). The first is farm gate sales where farmers sell low volumes of livestock to roaming livestock traders. The second tier involves farmers or roaming traders selling cattle to relatively large cattle traders at the primary or local livestock markets. The third tier involves livestock traders from primary markets selling relatively large volumes of cattle to secondary markets. Long distance livestock traders typically buy large volumes of cattle from secondary markets and sell them to cities or districts within or outside the country. At each tier, the price of cattle is guided by the biophysical characters of the animal, transport costs, knowledge of the demand and supply situation and negotiation skills (22, 23).

In Uganda, the predominant livestock production system in eastern and northern Uganda is smallholder mixed farming (24). The main reason for keeping cattle in the smallholder mixed farming systems of eastern and northern region of Uganda is to provide draft power for crop cultivation with work oxen representing 36.5– 43.7% of the cattle population (25, 26). The purpose for which livestock are maintained influences livestock trade as well as herd structure and size. Herd structure and size determine availability of animals for breeding, sale and plowing. Surpluses and deficits result in inter-district livestock trade (27). Individual cattle herds are composed of animals of different ages, sexes, and function reflecting diverse production systems. Parameters such as herd growth rate, milk yield, draft power output, fertility, and mortality (28) can be used to project future herd sizes, structures, and offtake of animals (for sale or slaughter) under different production systems. Forecasts require comprehensive data for herd age structure, age-specific reproduction, mortality, and offtake rates to enable herd modelling (29). Models applied for African animal trypanosomiasis (AAT) include a bio-economic simulation model with economic surplus (30), dynamic herd models looking at meat and milk outputs (31, 32), and draft power (33). The model described by Shaw (34) differs by not only incorporating productivity of draft cattle but also computing the number of work oxen that need to be “imported” into the herd depending on herd structure and local requirements.

Few studies have attempted to evaluate critical linkages between livestock production system, livestock spread, and potential spread of zoonotic diseases where cattle are disease reservoirs. This study aimed to identify and evaluate the critical economic linkages and implications that may result in sustained transmission of rHAT at the farm (downstream) and livestock market (upstream) levels. This was achieved by (1) identifying and evaluating the key characteristics of the herd dynamics at the farm level including use of work oxen, (2) identifying and analyzing the main features of the livestock market, and (3) using cattle trade data for probabilistic rHAT risk analysis associated with moving cattle from eastern to northern Uganda. Although most livestock diseases can be spread along the value chain, HAT was chosen in this study due to its public health implication. However, the approach and methods used in this study is applicable to most livestock diseases.

## Method

### Study Area and Design

**Figure 1:**
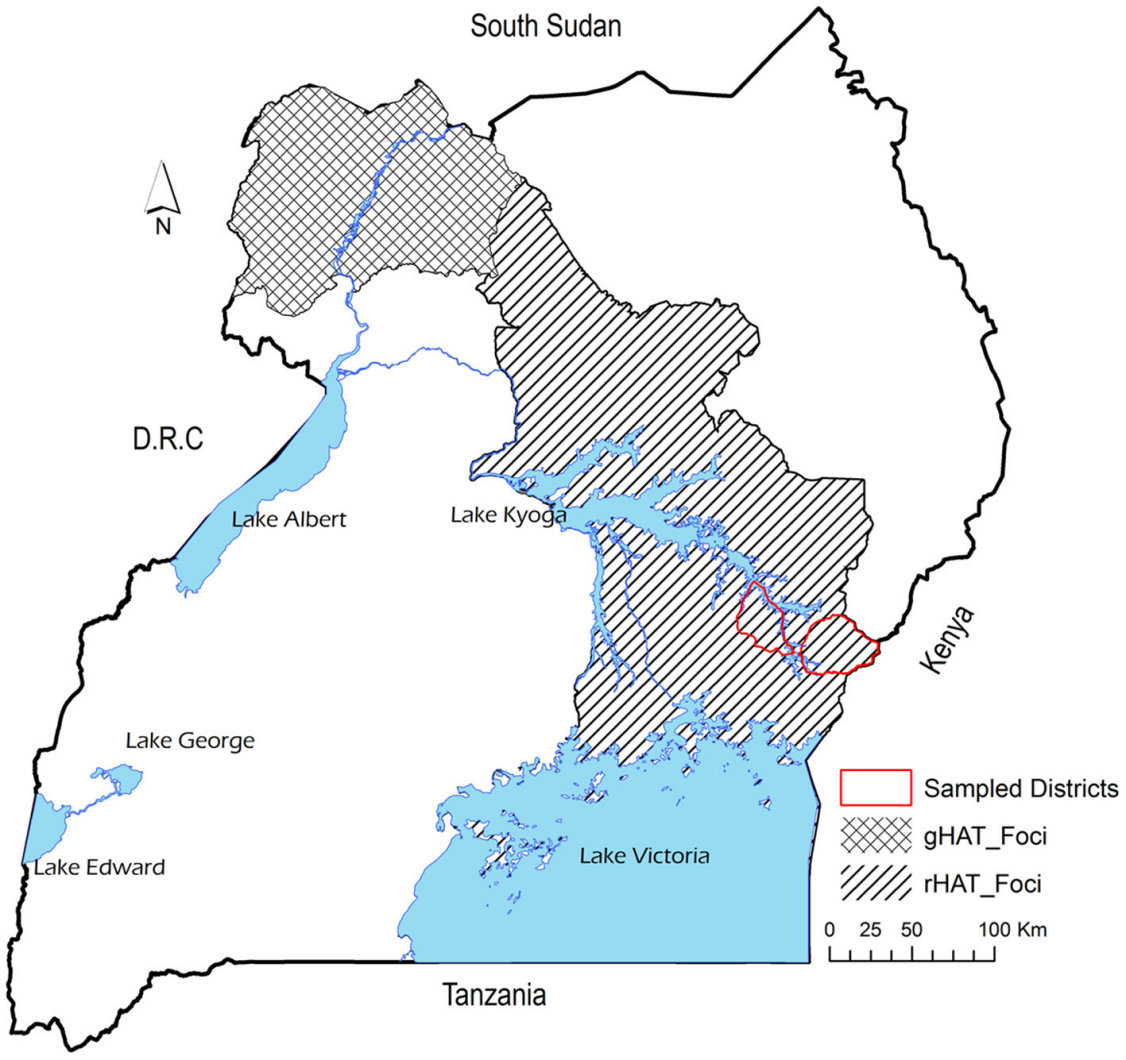
Map showing the study area and the rHAT and gHAT foci.

**Figure 2:**
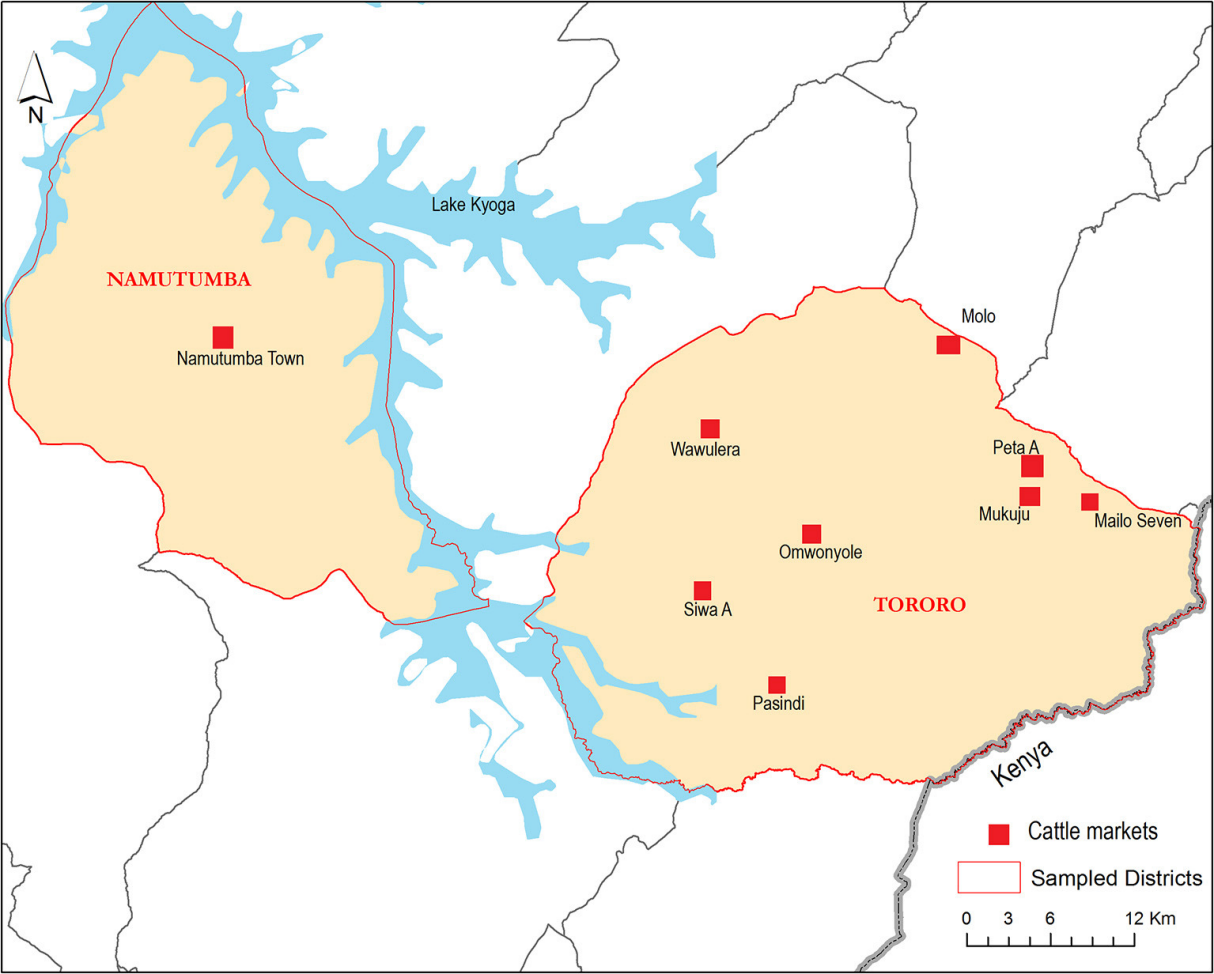
Map showing Tororo and Namutumba Districts and where the cattle market data was collected (in red). Water bodies are shown in blue.

The study was conducted in Tororo and Namutumba districts, south-east Uganda between 20^th^ to 28^th^ October 2014 to collect datasets representative of live cattle trade in semi-arid areas of eastern and northern Uganda. Figure 1 shows the study area and the HAT foci and Figure 2 shows the cattle markets where data was collected. Animal African trypanosomiasis (AAT) and zoonotic rHAT are endemic in eastern Uganda (35–37). The types of livestock drugs available and utilized in south-east Uganda for disease control in livestock have been previously explored (38). Equally, the distribution of tsetse fly eastern and northern Uganda has been described in other studies (39–41). Smallholder mixed crop-livestock production systems are the predominant agricultural system in Uganda where cattle production is geared towards supporting traction (25). According to (26), the draft cattle start work at 2.6 years until they are 11.1 years, resulting in a working life of 8.5 years. Farmers spend about 18 months training young males for draft reaching optimal efficiency at 4.1 years. The main plowing season is March and April during the long rainy season and October during the short rainy season.

**Figure 3:**
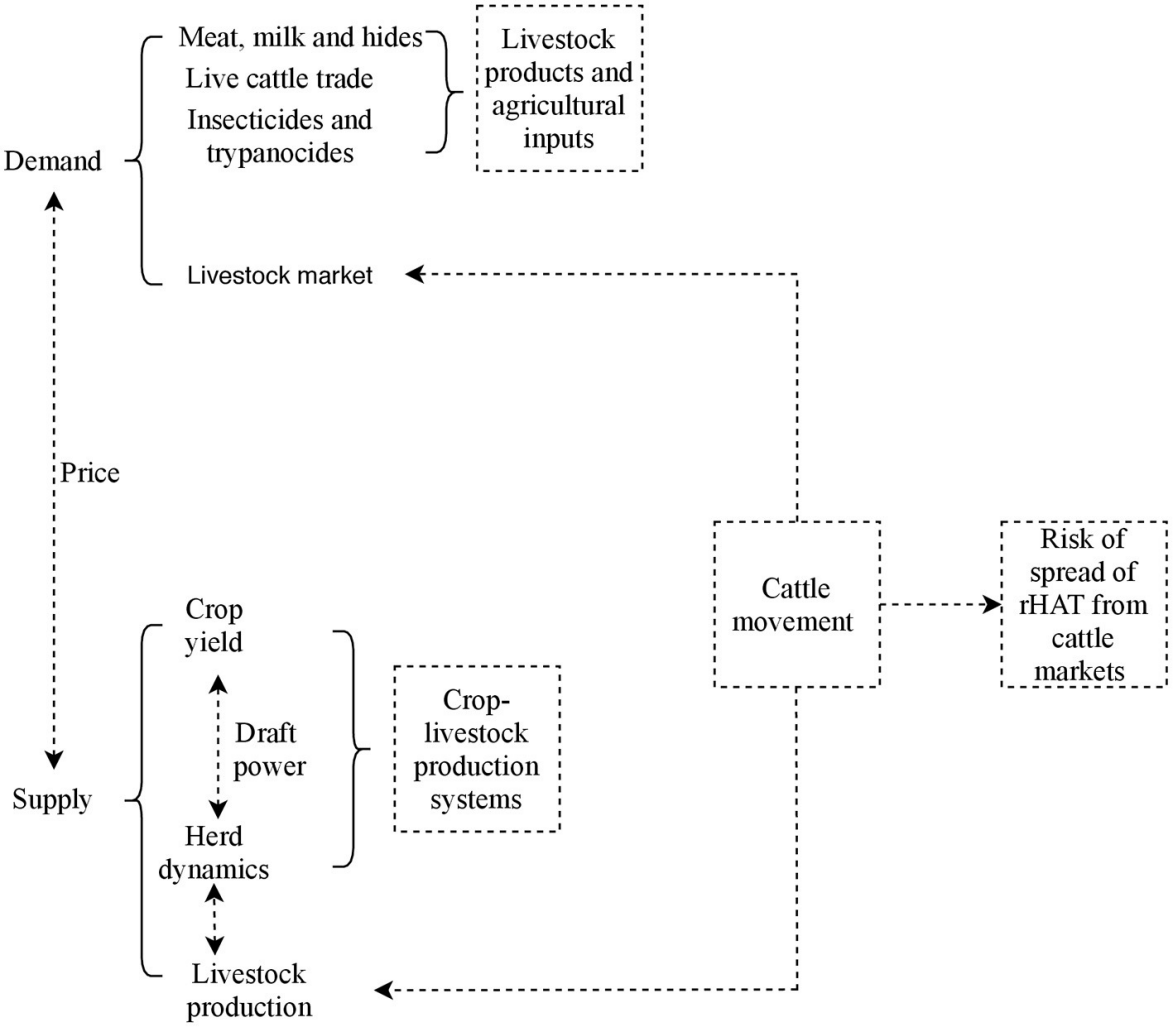
Linkages between livestock production, market and spread of rHAT.

Tororo and Namutumba, which provide insights on herd structure and livestock marketing in crop-livestock production systems in Uganda, experience two dry seasons between June to August and December to February and the rainy season is from March to April and October to December (39). The study area has extensively been described elsewhere (42). The conceptual model used in this study is shown in Figure 3. Key components studied were herd dynamics at the farm (downstream), live cattle trade (upstream), and risk of rHAT spread from eastern to northern Uganda due to herd dynamics and cattle trade.

### Data Collection and Analysis of the Livestock Markets

To understand upstream economic activities, a list of all live livestock markets in Tororo and Namutumba districts was obtained from the records available at the district veterinary office in 2014. According to the list obtained from Tororo district veterinary office, the cattle markets included Siwa A (also known as Siwa), Peta parima (also known as Peta A), Mairo seven, Pasindi, Munyole (also known as Omunyole), Molo, Mukuju and Wawulera. Namutumba was the only cattle market in Tororo district. All the cattle traders (i.e. census survey method), through verbal consent, were interviewed using semi-structured interviews. The interviews captured: (i) interviewee information, (ii) the livestock markets where cattle traders mostly sourced their cattle from in the whole annual livestock trade cycle, (iii) the livestock markets where these cattle were mostly sold to, (iv) the number of cattle each trader brought to the livestock market, (v) peak periods of sales per year, (vi) number and age/sex of cattle traded per month during each peak period (using a 12 month recall), and (vii) the time taken to sell the cattle. The number of cattle sold was obtained from livestock movement permit register. Livestock markets were visited on their respective market days and the following parameters were recorded: (i) number, age and sex of cattle present, (ii) number of cattle sold, (iii) the frequency of cattle trade activities, and (iv) number of local animal health providers present. Semi-structured interview questionnaires were administered to farmers who have come to purchase their cattle to establish where they mostly sourced their cattle from. Information from semi-structured questionnaire was entered and cleaned in Microsoft Excel. Focus group discussions with cattle traders, buyers and other traders in each market were undertaken and key informant interviews (43) involved discussions with local animal health providers and local council authorities. Local animal health providers were local government and private veterinarians and animal health assistants, while local council authorities were local council employees charged with the duty of collecting levy from livestock traders at the gate. Livestock data were analysed using the commodity supply chain approach (44, 45). The stages of analysis included the different tiers of livestock marketing *i.e*. farm gate to the large markets. At each stage along the chain, the approach permitted three types of analysis: costs and margins (price transmission analysis), flows (places, volumes, and directions), and the social relations of trade (46, 47). All the secondary data (from focus group discussions, key informant interviews and certain data sets within the questionnaire that had uncertainty, were modelled using Monte Carlo simulation with a 95% uncertainty interval (UI) in R statistical software version 3.2.2 (48).

### Parameters for Herd Modelling

To understand downstream economic activities, the bio-economic herd simulation model (34, 49) was used to simulate the effect of the herd structure on exportation and importation of cattle in the herd in eastern and northern Uganda. Parameters required for the herd model included: (i) cattle population, (ii) herd composition by age, sex and use for draft power, and (iii) off-take and mortality rates for these categories and calving rate. The cattle population used in the cattle herd model was obtained from the national livestock census (50). The cattle population used for eastern and northern Uganda was 2,488,467 and 3,921,849 respectively. The herd structure parameters were derived and averaged from (25, 26) given these studies were conducted in smallholder mixed production systems (agro-pastoral systems) in Uganda. The parameters used for the cattle herd model have been shown in Table 1. Based on the adult male draft cattle population of 28.5%, the required number of draft cattle in eastern and northern Uganda was 709,213 and 1,117,726 respectively and this was rounded off to 715,000 and 1,125,000 respectively to enhance model performance.

**Table 1:** Cattle herd composition and production parameters used for the herd model.

**Age, sex, and function**	**Starting herd composition in %**	**Mortality in %**	**Off-take in %**	**Calving rate in %**
Females aged 0 to 1	7.3	25.0	0.0	–
Females aged 1 to 2	5.8	8.0	0.0	–
Females aged 2 to 3	5.5	8.0	5.0	–
Females aged 3 to 4	5.3	8.0	5.0	–
Females aged 4 and over	27	8.5	9.0	50
Males aged 0 to 1	7.5	25.0	0.0	–
Males aged 1 to 2	6.3	8.0	2.0	–
Males aged 2 to 3	6.0	8.0	2.0	–
Males age 3 to 4	0.5	8.0	5.0	–
Males aged 4 and over	0.5	8.5	40.0	–
Work oxen aged 3 to 4	5.0	8.0	0.0	–
Work oxen aged 4 and over	23.5	9.5	13.0	–

To mimic the current herd composition and livestock keeper preferences, it was assumed that 95% of young male cattle were allocated to draft as calculated for high oxen use systems in (49). Additionally, an initial cattle population of 10,000 was used to assess the number of years, within 20-year projection, the herd model stabilized. It was found that the end of year herd size and growth rate stabilized after 5 years. Consequently, a 6-year projection, which included the base year, was used as a cut-off for this study instead of a 20-year projection.

The number of draft cattle imported was computed by first setting the number of draft cattle required for each year; then subtracting this from number of draft cattle available locally (young males reaching the age when they start to work and adult draft males, adjusted for mortality and offtake). This estimated the deficit which will need to be met by bringing in cattle from outside the area. The model outputs gave the changing numbers year by year, and thus the growth rate and herd composition.

### Cattle Herd Model Assumptions and Validation

It was assumed that the mortality, calving and offtake rates would remain constant over the 6-year projection period. Mortality, calving, and offtake rates affect the herd size, herd structure and, this may change due several factors such as farmers deciding to purchase other types of cattle (i.e. de-investing in animal traction), cattle prices, implementation of disease control programs among others. Such factors are not possible to capture using bioeconomic herd modelling hence mortality, calving and offtake rates were fixed. Furthermore, one of the interests of this study was to identify deficits in the herd structure and how such deficits can be met to maintain the current herd size. It was assumed that farmers would aim to maintain their herd structure and size. Validation of the model was done by checking the consistency of results and comparing results with different inputs (rationalism method), tracing of attributes over the project 6-year period, and scrutiny of all input parameters (face validity method) (51). This included reducing the calf mortality from 25 to 20% as well as increasing calving rate 50 to 55% as part checking model performance and sensitivity analysis. It was expected that the number of draft male cattle imported would reduce when the calf mortality and calving rate were reduced and increased respectively if the cattle herd model was accurate. External validation was not possible because of the lack of district level livestock census data covering 2014 to 2020. However, according to national census, the cattle population in Uganda reduced by 1.2% between 2016 and 2017, although the overall cattle population between 2008 and 2018 was expected to increase by 3.0% per annum in Uganda (52).

### Estimating Risk of Disease Spread

To understand the implications of cattle trade and herd dynamics, the risk of rHAT spread from eastern to northern Uganda was estimated. The risk of invasion, denoted as *p*_*inv*_, was defined as the probability of rHAT being transmitted to northern region (denoted as *p*_*n*_) through cattle trade from a disease-endemic eastern region of Uganda (denoted as *p*_*e*_) within a one-year period. Given that rHAT does not clinically affect cattle and that routine surveillance for animal diseases is not done at the cattle markets before cattle is moved (17), we assumed that the disease would be able to be spread from eastern to northern Uganda without detection. The probability of exporting a diseased animal from eastern to northern Uganda (denoted as *p*_*i*_) was estimated by the rHAT prevalence in cattle markets in eastern Uganda i.e. rHAT endemic region. It has been reported that the average rHAT prevalence in cattle markets in eastern Uganda is 1.5% (16), hence the probability of exporting rHAT from endemic region used in this study was 0.015. The estimated annual number of cattle moved from eastern to northern Uganda, denoted as *m*_*e, n*_, was estimated from information collected from cattle traders. This involved determining the number of cattle markets in eastern Uganda from which cattle were moved to the northern region as mentioned by the cattle traders. This information provided the probability of purchased cattle being moved from eastern to northern Uganda, denoted as *p*_*m*_. Additionally, using uniform distribution and 10,000 simulations in R software (48), the minimum and maximum number of cattle sold as obtained from the livestock movement permit register was used to estimate the average number of cattle that would be purchased in cattle markets that moved cattle from eastern to northern Uganda. Use of minimum and maximum number of cattle sold was important as it covered any potential seasonal changes in cattle sales.

Assuming homogenous mixing of cattle and full susceptibility, introduction of rHAT in non-endemic areas of northern Uganda may lead to either the disease being spread or fading out depending on the basic reproduction number, denoted as *R*_0_. The *R*_0_ for rHAT was obtained from past studies (53, 54); average *R*_0_ of 1.287 was used in this study. Also, with *R*_0_ being the potential of rHAT to spread within a population in eastern and northern Uganda, the possibility of rHAT fading out soon after introduction of one affected cattle from eastern into northern Uganda can be denoted as 1/*R*_0_ (55) and the probability of a prolonged outbreak (*p*_*o*_) can be denoted as 1−1/*R*_0_. Using the approach in (56), the risk of rHAT invasion (*p*_*inv*_) from cattle reservoir in northern Uganda was computed as:

$$\begin{array}{l} p_{inv}=1-{\left(1-p_{i}p_{o}\right)}^{m_{e,n}} \end{array}$$

## Results

### Livestock Market Dynamics

Nine livestock markets were visited, and 197 cattle traders and 113 farmers (who were buying cattle) were interviewed. Livestock markets are managed by the local council and cattle traders were taxed a standard fee of United States dollar (US$) 0.5 for a movement permit. There were two levels of cattle markets namely primary and secondary. Secondary level cattle markets in Tororo district included Siwa A, Peta parima (also known as Peta A), Mairo seven, Pasindi, Munyole (also known as Omunyole), Mukuju and Wawulera. Molo was the only primary level cattle market in Tororo district. Namutumba was also a secondary cattle market.

**Figure 4:**
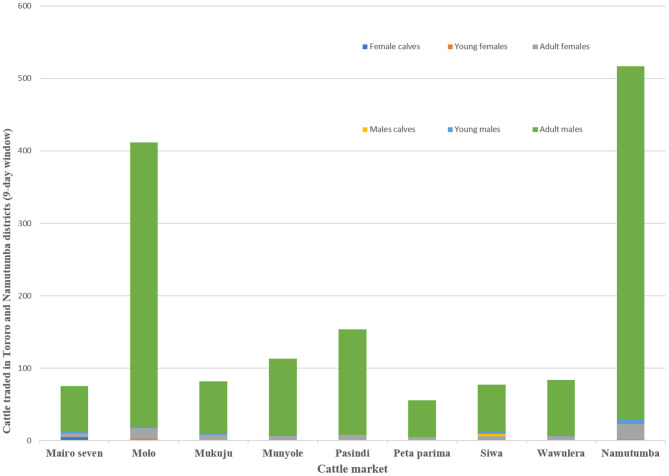
Cattle traded in Tororo and Namutumba Districts (9-day window).

Focus group discussions and key informant interviews indicated that outside of Tororo District, Namutumba and Soroti were primary level cattle markets. The size of the livestock market varied depending on holding capacity and number of cattle traded with the largest markets being the primary markets (i.e. Namutumba and Molo). Larger markets traded cattle and other livestock including poultry and goats. Smaller secondary livestock markets (i.e. Pasindi, Siwa, Munyole and Peta Parima) only traded cattle. Each market operated once a week with traders attending on a rotational basis. Cattle imported into Tororo district originated from Namutumba (47.0% respondents), Soroti (42.0% respondents), Lira (6.0% respondents), and Mbale (5.0% respondents). During the 9-day visit to all the cattle markets, there were a total of 1,565 cattle. Figure 4 shows the number and types of cattle traded. The number of cattle sold in Mairo seven, Molo, Mukuju, Munyole, Pasindi, Peta parima, Siwa, Wawulera, and Namutumba was 28, 131, 29, 27, 46, 17, 22, 25, and 189 respectively (minimum: 17, maximum: 189, standard deviation: 60).

According to data obtained from the semi-structured interviews, 76 out 197 cattle traders mentioned that they mostly moved cattle from eastern to northern Uganda. Therefore, the probability of moving (*p*_*m*_) from eastern to northern Uganda was estimated to be 0.385. The cattle markets from which cattle were sourced from in eastern Uganda and moved to northern region were seven and this included Kaberamaido, Soroti, Namutumba, Molo, Katakwi, Ngora, and Kumi. Also, the study found that most of the cattle from the seven cattle markets were moved to Lira, Adjumani, Dokolo, Gulu, Oyam, and Amuru in northern Uganda.

Interviews with 197 cattle traders showed a pattern of trade seasons during which cattle were traded annually depending on type. The cattle traders indicated that the first cattle trade season ran from January to March and predominantly included sale of young males (61.4% of the respondents) just before the plowing season in March and April. The second season from April to September involved the sale of mixed types of cattle (65.9% respondents) and a third season from October to December described sale of mostly culled old animals (81.0% respondents). The estimated total number of cattle traded annually as mentioned by cattle traders was 3,763 with each trading 19.1 cattle annually. Table 2 summarizes the annual cattle traded per trader in each season.

**Table 2:** Annual average number of cattle traded per trader (*n* = 197).

**Annual cattle trade season based on time period**	**Annual number and type of cattle traded per trader (mean and SD)**						
	**Female calves (0–1years)**	**Young females (1–4 years)**	**Adult females (1-4 years)**	**Male calves (0-1 years)**	**Young males (1-4 years)**	**Adult males**	**Total number cattle traded**
January to March	0.0 (0)	0.5 (0.9)	0.3 (0.9)	0.0 (0)	4.7 (3.1)	1.2 (1.9)	6.7 (3.2)
April to September	0.0 (0)	0.6 (0.9)	0.4 (0.6)	0.0 (0)	1.7 (1.1)	2.0 (1.1)	4.7 (2.1)
October to December	0.0 (0)	0.0 (0)	0.3 (0.6)	0.0 (0)	0.0 (0)	7.4 (4.1)	7.7 (4.2)
Total	0.0	1.1	1.0	0.0	6.4	10.6	19.1

Focus group discussions revealed that traders who mostly came from Lira, Arua, Moyo and Kotido districts in northwest Uganda bought young males from south east Uganda and sold them to non-government organizations who train work oxen for plowing. Afterwards, livestock traders buy back these animals after one and half years of training for draft work and sell them back to farmers in south east Uganda at a higher price. Traders typically bought one and half year old young males at US$ 71.2 (95% UI: 54.7, 89.1) and sold them back to farmers in Tororo district at US$ 224 (95% UI: 182.7, 267.2) when the cattle were three and half years of age, making an average gross gain of US$ 122.8 (summarized in Table 3).

**Table 3:** Price of cattle in the livestock markets by age and sex.

**Type of cattle by age/sex**	**Mean price bought (95% UI)**	**Mean price sold (95% UI)**	**Gross profit (in USD)**
Calves in Tororo district	23.5 (18.2–28.5)	37.8 (36.1–39.5)	14.3
Untrained young males destined for draft work bought from farmers in south-east and sold to NGOs in northwest Uganda	71.2 (54.7–89.1)	90.3 (87.4–92.3)	19.1
Trained young draft males bought from NGOs in northwest and sold to farmers in south-east Uganda	101.4 (98.6–104.7)	224.2 (182.7–267.2)	122.8
Young females in Tororo district	48.6 (43.5, 53.7)	108.1 (90.7–125)	59.5
Cows in Tororo district	162.1 (144.4–179.0)	207.7 (181.6–232.5)	45.6
Adult males in Tororo district sold for draft or slaughter	269.2 (252.7–286.8)	381.0 (275.8–495.2)	111.8

Interviews were also conducted with the 113 livestock buyers in the livestock markets. Of the 113 farmers interviewed, 4.4% acquired their cattle through inheritance, 69.9% from cattle markets, 10.6% through buying from neighbors, 7.9% through restocking programmes, and 7.0% from exchange of goats for cattle.

### Cattle Herd Dynamics

**Figure 5:**
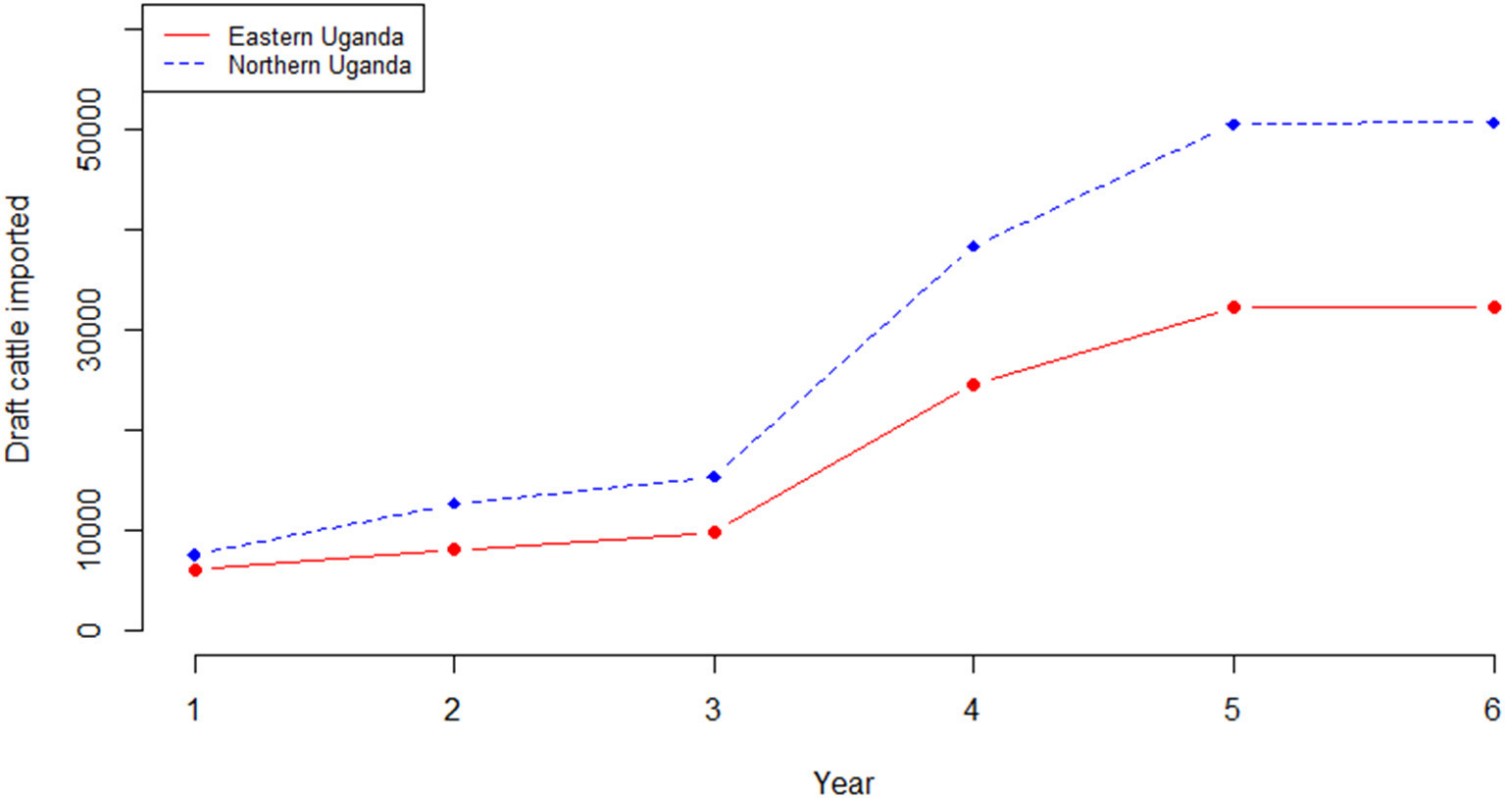
Simulated number of draft cattle imported over a 6-year period.

Outputs from the herd model, which is an extrapolation of the current trends in livestock production within smallholder mixed farming systems showed, that the cattle herd size would fall from 2,488,467 in the base year to 2,062,340 at the end of the 6-year projection period in eastern Uganda if all factors (i.e. use of cattle, disease prevalence, herd structure and size) remained the same. Equally, in northern Uganda, the cattle herd size would fall from 3,921,849 to 3,248,722 at the end of the 6-year simulation period. For both regions, the cow adult male ratio in the base year was 1.1 falling marginally to 1.0 by the end of the 6-year period. Also, for both regions, the annual average herd growth rate was−3.2% over the projected 6-year period. In eastern Uganda, the estimated number of draft cattle imported to bridge the deficit was 5,991 in year 1 rising to 32,239 at the end of the 6-year projection period; draft cattle would be imported at an average of 18,826 annually over the same period. In northern Uganda, 7,529 draft cattle need to be imported in year 1 rising to 50,494 in year 6; an average of 29,164 draft cattle annually. Figure 5 show the simulated draft cattle imported in eastern and northern Uganda. The projected herd structure at the end of the 6-year for both eastern and northern Uganda has been shown in Table 4. The projected herd structure for the two agro-pastoral regions indicates that male cattle will still be the predominant sex with 45 and 35.2% being draft cattle in eastern and northern Uganda respectively as shown in Table 4.

**Table 4:** Projected cattle herd composition for the 6-year period.

**Age category (in years)**	**Eastern uganda**		**Northern uganda**	
	**Female (%)**	**Male (%)**	**Female (%)**	**Male (%)**
0–1	5.3	5.9	6.4	6.9
1–2	4.1	4.6	4.9	5.4
2–3	4.1	4.4	4.8	5.2
3–4	3.7	0.2	4.3	0.2
4+	22.3	0.4^*^	26.3	0.4^*^
Draft male 3–4	—	8.5	—	6.7
Draft male 4+	—	36.5	—	28.5
Total	39.5	60.5	46.7	53.3

* *Non draft cattle*.

As part validation, the herd model was re-run with lower calf death rates (20.0% for females and 17.0% for males) and a higher calving rate of 55%. In eastern Uganda, the number of draft males imported was 5,991 in year 1 increasing to 15,135 by year 6 as opposed to 32,239 when the original parameters were used. In northern Uganda, the estimated number of draft cattle imported increased from 7,529 to 23,562 by the end of year 6 compared to 50,494 when the original parameters were used. These changes were expected, i.e. lower calf mortality and high calving rate increases the number of draft cattle in the farm which in turn result in reduced importation. Therefore, this indicated that the model worked well.

### Risk of Disease Transmission

Using minimum and maximum number of cattle sold (i.e. mean of 102 per cattle market), the number of cattle markets where cattle traders mostly sourced their animals from in eastern region (i.e. seven in total) and moved them to northern region, and *p*_*m*_ of 0.385, it was estimated that the number of cattle moved to northern Uganda from eastern Uganda (i.e. *m*_*e, n*_) was 14,294 per year. The probability of a prolonged outbreak (i.e. *p*_*o*_) was estimated to be 0.222. Consequently, the potential risk of rHAT invasion and spread from eastern into northern Uganda through cattle trade was estimated to be 1.

## Discussion

Livestock movement and implications for disease spread is well described in developed countries where data is usually available (57–61). In developing countries like Uganda, data on livestock movement is scarce resulting in limited capacity to routinely monitor diseases. Additionally in developing countries, the link between livestock production and potential risk of spread of diseases has not been well understood. In Uganda, past studies have shown that cattle restocking as part of post conflict recovery program, is one of the reasons for cattle movement from eastern to northern Uganda (16). Our study quantitatively captures the the potential rHAT risk of spread between eastern Uganda where the disease is endemic, and the disease free northern region via cattle cattle trade for the first time. Also, for the first time, this study examines the reasons behind the cattle trade in smallholder mixed farming systems (i.e. agro-pastoral systems) in developing countries like Uganda apart from restocking efforts. The link between on-farm livestock production and livestock trade is important in understanding the potential risk of disease spread for disease control pruposes.

This study showed that livestock trade is complex involving multiple layers of transaction. The first layer involved sourcing of cattle by small scale traders from household to household and sale of cattle by the farmers to the primary markets. The second layer mostly involved small and large scale traders. Farmers did not participate in cattle trade as they did not have market information nor the negotiation capacities required for sale of cattle. Once cattle traders acquired the animals, the majority of them moved the cattle on foot or on trucks for months from one market to the other and back depending on the season. Long distance cattle trade using trucks may result in spread of rHAT northwards as it increases the volume, distance, and frequency of inter-district trade. Additionally, walking of cattle from market may also expose them to tsetse infestation and thereby spread of rHAT within and between districts.

Cattle trade in south-east and northern Uganda showed three distinct patterns (1) a short and high volume trade in young males just before the plowing season, (2) a protracted and moderate volume trade in mixed types of cattle, and (3) a short and high volume trade in culled old cattle. Sales patterns may be attributed to a high demand for young males during the plowing season and high demand for disposed old draft cattle for slaughter during the festive season in December. Continous trade and movement of cattle in high volumes particularly during the rainy season in March and December may have potential epidemiological implications. The risk of exposure of large volumes of cattle to tsetse flies during and after the rainy season is high. Tsetse fly density is higher during and after rains when the ground is wet and the ambient temperature is right. The newly emerged tsetse flies (teneral flies) are most susceptible to becoming infected with trypanosomiasis (5).

The cattle supply chain analysis demonstrated that the cattle market in agro-pastoral areas is dominated by trade in draft and adult male cattle. Supply and demand of cattle was based on the cattle herd structure with farmers importing draft cattle due to deficits as revealed by the cattle herd model. Similar observations have been made in Madagascar, where cattle trade is based mostly on young males for draft work and sale of old draft cattle for slaughter (62). Therefore, cattle herd structure plays a crucial role in influecing the type of cattle sold in the cattle market as well as the pattern of livestock movement and ultimately the potential risk of disease spread. Additionally, if mortality and calving rates in eastern and northern Uganda remain unchanged, then the need for importing draft cattle will continue with increasing numbers being imported over the coming years resulting in increased cattle movement and risk of spread of rHAT in Uganda. However, in the pastoral production systems which dominate certain parts of northern and eastern Uganda, the herd structures are dominated by female cattle as build up of the herd size is the main aim of cattle keeping (63).

Analysis of the potential risk of rHAT spread through cattle trade showed that even at a low rHAT cattle prevalence of 1.5% in eastern Uganda and low transmissiblity with an *R*_0_ of 1.287, the probability of the disease invading and spreading in northern Uganda was high (100%). Similar observations have made in West Africa where a hypthetical disease of less than 1% prevelance and *R*_0_ of 1.25 would have a high probability of an outbreak (80%), and if the prevalence is between 1–10% then the probabaility of an outbreak is 100% through cattle trade (56). However, outbreak of rHAT in humans in northern Uganda would depend on several factors such as tsetse fly density, human-cattle interactions, host characteristics, and human migration among others. The high risk of rHAT spread from eastern to northern indicates that routine disease surveillance is required in both regions. From the data obtained in this study, it is possible that primary markets in eastern Uganda may play a major role rHAT transmission as they acted as congregation points of cattle with cattle being moved long distance using trucks. To be cost effective, rHAT surveillance can be targeted to the primary markets in eastern Uganda (e.g. Namutumba, Molo, Soroti, Katakwi, Kumi, and Ngora) and those markets in northern Uganda that received most of the cattle (e.g. Lira, Adjumani, Dokolo, Gulu, Oyam, and Amuru). As part of routine disease control, it may be essential to use cost-effective methods such as restricted application protocol and curative trypanocides at the point of sale (64).

Still in relation to potential rHAT spread, movement of young male cattle from south-east Uganda northwards for training in draft work and better finacial returns may drive infection northwards. This is because the young male cattle would spend around two and a half years in northern Uganda, mostly Lira, Arua, Kotido and Moyo districts. This is a sufficient period to spread *T. b. rhodesiense*. Although, tsetse flies prefer to feed on larger cattle (65, 66), it has been reported that cattle which are over 18 months of age were more likely to be infected with *T. brucei sensu lato* (67). To prevent movement of young male cattle from eastern to northern Uganda, an animal traction training program can be provided in the eastern region as an incentive to reduce the risk of rHAT spread.

The approach of identifying critical economic and epidemiological linkages that may result in sustained spread of rHAT is not without limitations. Ideally more livestock markets would have been visited with a more extensive geographical distribution. Second, a cross sectional survey as used in this study cannot fully capture seasonal variations or annual economic activities. Third, the movement of cattle was extrapolated from cattle traders and this may result in over or understimation. Fourth, the data captured was only from formal cattle markets. Informal trade occurs in Uganda and therefore the study may underestimated the scale of cattle trade. Fifth, more rigourous rHAT risk analysis studies is still required given the uncertainty in the data collected and the sample size of this study. Sixth, cattle herd parameters as used in this study can change due to various reasons such as drought and disease outbreaks and this could result in changes in livestock numbers and herd structure. Based on the limitations, the study recommends future longitudinal studies that aim to sample more livestock markets, incorporating epidemiological data on livestock diseases.

## Conclusion

This study found that there is an intrinsic and intricate link between cattle herd dynamics and livestock trade. It is this link that result in livestock movement and ultimately potential spread of rHAT and perhaps other diseases. Equally, there is a high probability that rHAT may spread from eastern to northern Uganda via cattle trade in a scenario where there is an original outbreak in the eastern region with demand for draft cattle playing a major role in this. The spread of rHAT northwards may result in a public health crisis given the difficulties in distinguishing between the disease and rHAT; the two diseases also require different treatment. Given the high likelihood of rHAT spread northrwards, hence the mergence with gHAT, this study recommends enhanced disease surveillance and control.

## Data Availability Statement

The original contributions presented in the study are included in the article/supplementary material, further inquiries can be directed to the corresponding author/s.

## Ethics Statement

The studies involving human participants were reviewed and approved by the Makerere University College of Veterinary Medicine Animal Resources and Biosecurity ethical review board, and by the Uganda National Council for Science and Technology (approval number HS1336). The patients/participants provided their written informed consent to participate in this study.

## Author Contributions

WO was responsible for conception, design, collection, drafting and analysis of data. DM was involved in conception of the study and data collection. AS was involved in conception, design, analysis and drafting the manuscript. SW and CW were involved in conception of the study design and revision of the manuscript. EM was involved in design and drafting of the manuscript. All authors read and approved the final version of the manuscript.

## Conflict of Interest

AS was employed by AVIA-GIS. The remaining authors declare that the research was conducted in the absence of any commercial or financial relationships that could be construed as a potential conflict of interest.

## Publisher's Note

All claims expressed in this article are solely those of the authors and do not necessarily represent those of their affiliated organizations, or those of the publisher, the editors and the reviewers. Any product that may be evaluated in this article, or claim that may be made by its manufacturer, is not guaranteed or endorsed by the publisher.
